# Prevalence, Intensity, and Risk Factors of *Schistosoma mansoni* and Other Intestinal Parasites: A Cross-Sectional Study in Rural Agricultural Communities of Lango Subregion, Uganda

**DOI:** 10.1155/japr/8867072

**Published:** 2025-10-11

**Authors:** John Paul Byagamy, Robert Opiro, Margaret Nyafwono, Geoffrey Maxwell Malinga, Richard Echodu, Emmanuel Igwaro Odongo-Aginya

**Affiliations:** ^1^Department of Environment and Natural Resources Management, Faculty of Agriculture and Environment, Gulu University, Gulu City, Uganda; ^2^Department of Biology, Faculty of Science, Gulu University, Gulu City, Uganda; ^3^Department of Microbiology and Immunology, Faculty of Medicine, Gulu University, Gulu City, Uganda

**Keywords:** intestinal helminth parasites, Lango subregion, northern Uganda, rural agricultural communities, *Schistosoma mansoni* infection

## Abstract

**Background:**

In developing countries such as Uganda, intestinal parasitic infections and schistosomiasis remain a serious threat to public health. Local epidemiological data are critical for the prevention and control of the disease. This study was aimed at determining the prevalence, intensity, and risk factors of schistosomiasis and other intestinal parasite infections among rural agricultural communities in the Lango subregion, northern Uganda.

**Methods:**

A cross-sectional study was carried out in Lira city, Kole, and Lira district in the Lango subregion, northern Uganda, involving 438 participants aged between 5 and 85 years. Urine and stool samples were collected and examined for parasites using urine circulating cathodic antigen (POC-CCA), filtration methods, and the Odongo-Aginya technique. Information on sociodemographic factors and other predisposing factors to infections was obtained using questionnaires. Descriptive analyses using frequency and percentages were used to summarize the independent and dependent variables. Pearson's chi-square test and multivariable logistic regression models were used to determine the association between *Schistosoma mansoni* infections and risk factors.

**Results:**

The overall prevalence of parasitic infections using the Odongo-Aginya method was 120/438 (27.4%). *S. mansoni* was the dominant parasite with 93/438 (21.2%), and *Ascaris lumbricoides* (2.5%), *Entamoeba complex* (0.9%), *hookworms* (0.7%), and *Entamoeba coli*, *Enterobius vermicularis*, and *Diphyllobothrium latum* (0.5%), respectively, and *Trichuris trichiura*, *Fasciola hepatica*, *Giardia lamblia*, *Strongyloides stercoralis*, *Paragonimus westermani*, and *Trichostrongylus ova* (0.2%). *Schistosoma haematobium* was not detected in the study. There were light, moderate, and heavy infections with *S. mansoni* among the participants with 66 (72%), 24 (26%), and 2 (2.2%), respectively. According to multivariable logistic regression analysis, participant age (AOR = 1.448, 95% CI: 1.207–1.737, *p* < 0.001) and subcounty location (AOR = 1.837, 95% CI: 1.274–2.650, *p* < 0.001) were significantly associated with *S. mansoni* infection.

**Conclusion:**

The study reveals that *S. mansoni* infections are still highly prevalent in Lango subregion, with age and subcounty location as major risk factors. The findings highlight an ongoing public health challenge, particularly among high-risk age groups and in specific localities. Targeted interventions, such as sustained mass drug administration of at-risk population groups and locations, improved access to safe drinking water, and community health education, are essential to reduce infection rates and improve overall well-being in affected areas.

## 1. Background

Human schistosomiasis (bilharziasis) is a water-borne parasitic disease caused by schistosomes and transmitted by snail intermediate hosts mainly of the genera *Biomphalaria* and *Bulinus* species [1]. Globally, it is estimated that about 252 million people are infected with *Schistosoma* species in 78 tropical countries and that 500–600 million people are at risk of infection [2]. Out of over 200 million cases of infection in a year, 120 million are symptomatic and 20 million have severe debilitating disease [3, 4]. The disease is commonly found in tropical countries with permanent water bodies infested with freshwater snail intermediate hosts that contain the infective cercariae in Asia, Africa, and South America [4, 5]. Both intestinal and urinary schistosomiasis cause acute and chronic diseases [4]. Infections occur during water contact activities along the permanent water bodies and in people engaged in domestic, agricultural, and recreational activities, which expose them to cercarial-infested water [6]. Recent estimates indicate that at least 290.8 million people require praziquantel treatment for schistosomiasis. However, only 97.2 million have been treated [7, 8]. The World Health Organization (WHO) has embarked on the elimination of schistosomiasis as a public health problem in endemic countries in a roadmap from 2021 to 2030 [9].

In sub-Saharan Africa, recent estimates reveal that 54 million people are currently infected with *Schistosoma mansoni*, causing blood in stool in 4.4 million, hepatomegaly in 8.5 million, and an annual mortality of 130,000 people [10]. Agricultural activities, mainly in areas of water resource development like irrigation fields and the construction of dams, have encouraged the spread of the disease [11, 12]. These project areas are suitable habitats for snail intermediate hosts shedding the cercariae, which are the infective stages of the parasite [11, 12]. A typical example of irrigation schemes' role in the transmission of schistosomiasis is in Egypt and Sudan, where the prevalence of periportal fibrosis is considerably high at 79.3% compared with countries such as Kenya and Mali, where the prevalence of periportal fibrosis is markedly low at 27.5% and 23.6%, respectively [13].

In Uganda, schistosomiasis causes high morbidity and mortality in most communities, especially along the lakeshores and river banks [4, 14, 15]. It is the second most significant disease in terms of public health impact and socioeconomic burden, following malaria [16, 17], causing both intestinal and urinary complications that significantly affect the quality of life [4, 18]. Countrywide, 81 districts and an estimated 5.4 million people are affected, with 14.7 million at risk of the infection [18]. The disease mainly affects the rural poor, especially peasant farmers. Infections do occur also among people living in urban or periurban settings [4, 19]. The WHO introduced the mass drug administration (MDA) in Uganda in 2003 as a way of reducing morbidity and mortality due to schistosomiasis in affected communities [19–22]. Despite this intervention, the prevalence is still high (more than 50%) among school children and rural communities [4, 23]. This group of people generally has low socioeconomic status with poor sanitation and limited access to clean water. Their main sources of income are fishing in the lakes or the rivers, swamps, dams, farming, and collection of water from these water bodies and, as a result, exposing them to infections [4, 19]. Furthermore, while schistosomiasis is a major focus of disease control programs, coinfection with intestinal parasites such as *Ascaris lumbricoides*, *Trichuris trichiura*, and hookworms, as well as *Entamoeba* species, is also widespread in the country [24, 25], with prevalence reaching as high as 90%, especially among children and rural communities with poor sanitation [4, 18]. These infections often share similar risk factors, including poor sanitation, contaminated water sources, and low socioeconomic status [26]. Coinfections with intestinal parasites can impact schistosomiasis treatment outcomes by altering immunological responses, contributing to malnutrition, anemia, and overall morbidity [27]. Individuals harboring multiple parasitic infections may respond differently to praziquantel and other anthelmintic therapies, necessitating an integrated approach to disease control [28].

The Lango subregion is known to be one of the areas harboring both *Schistosoma haematobium* and *S. mansoni* in Uganda [4, 24]. However, while previous studies have extensively documented *S. mansoni* prevalence in Uganda [4], there is a paucity of information concerning the actual magnitude of the disease and specific risk factors for infection in the subregion. Yet this kind of information is necessary to guide the prevention, control, and possibly elimination of the disease as suggested by WHO [4] and Adriko [9]. Furthermore, there is limited recent data on the burden of nonschistosome intestinal parasites in specific regions such as the Lango subregion [29]. Understanding their prevalence alongside schistosomiasis is crucial for designing comprehensive interventions that address multiple parasitic infections simultaneously.

This study is aimed at determining the prevalence, intensity, and risk factors of *S. mansoni* and other intestinal parasitic infections among rural agricultural communities in the Lango subregion of northern Uganda. By investigating both schistosomiasis and coendemic intestinal parasites, this study seeks to fill gaps in current epidemiological data and provide insights into how these infections interact within affected populations. The findings will inform targeted public health interventions, improved treatment strategies, and more effective control measures to reduce the burden of parasitic diseases in Uganda.

## 2. Materials and Methods

### 2.1. Study Area

**Figure 1:**
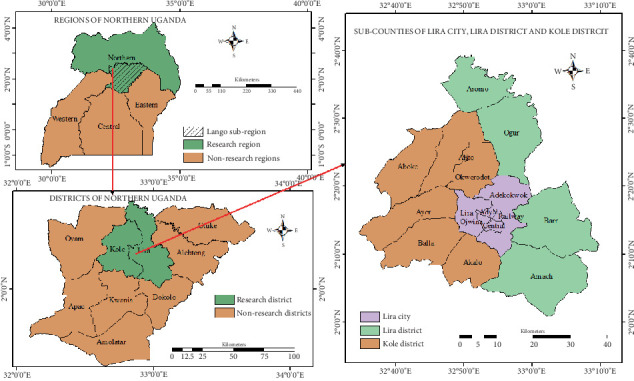
Location of study subcounties in the Lango subregion, northern Uganda.

The study was conducted in Lira (2°14⁣′50.0⁣^″^N 32°54⁣′00.0⁣^″^E) and Kole (2°23⁣′59.99⁣^″^N 32°47⁣′59.99⁣^″^E) districts in the Lango subregion, northern Uganda [30]. These districts are among the nine districts in the Lango subregion, predominantly inhabited by the Lango tribe (Figure 1). The study areas of Lira city, Kole, and Lira districts were selected due to their varied agroecological profiles, high agricultural activity, and historical burden of both *S. mansoni and S. haematobium*, making them representative of rural settings within the Lango subregion [30]. The main economic activities are commercial and subsistence farming, small-scale fishing, retail and wholesale business, and a commercial center for northern Uganda and the Lango subregion [30]. The area is characterized by a tropical type of climate with an average rainfall of 1500 mm, with the least received in January and the maximum in May [31]. There are two rainy seasons, April to May and August to November every year [31]. Temperature varies between 65°F and 95°F [31]. The dry seasons range from December to March and from June to August. Approximately 30% of the land area is covered by freshwater bodies like rivers, permanent and seasonal swamps, and wetlands. These water bodies are potential sources of infection for *S. mansoni* and *S. haematobium*, as they harbor numerous snail intermediate hosts [31]. The communities use these water bodies for different domestic purposes. The estimated total population of the Lango subregion is 2,513,700 people [32].

### 2.2. Study Design

The study was cross-sectional, targeting rural communities of Lango subregion, northern Uganda, to assess the prevalence of intestinal parasites, infection intensity, and risk factors of schistosomiasis. Participants were drawn from rural community members, especially farmers in wetlands, irrigation schemes, dams, and car washers, and screened for both *S. mansoni* and *S. haematobium*. Participants were subjected to both stool and urine examinations to detect and identify the schistosome eggs and other intestinal parasites.

### 2.3. Study Population

The target population comprised selected community members aged 5–85 years and all persons exposed to water containing snail vectors who were involved in washing clothes, wetland farming, rice fields, water collection for domestic use, and swamp car washers in all the selected districts in the Lango subregion.

### 2.4. Sample Size Determination

The sample size was calculated using the formula described by Lwanga [33]. *N* = *Z*^2^ × *PQ*/*d*^2^, where *N* is the desired sample size, *P* is the estimated prevalence, estimated to be 50%, *Q* is the percentage of people not infected (1 − *p*), *d* is the degree of precision required, usually set at 0.05, and *Z* is the confidence limit at 95% interval (1.96).

Substituting in the above formula, *N* = (1.96^2^ × 0.5 × (1–0.5) = 384.16 ≈ 384)/±0.05^2^.

However, after accounting for a 15% nonresponse rate, the final calculated sample size was 441, although only 438 participants provided both urine and stool samples.

### 2.5. Sampling Techniques

**Figure 2:**
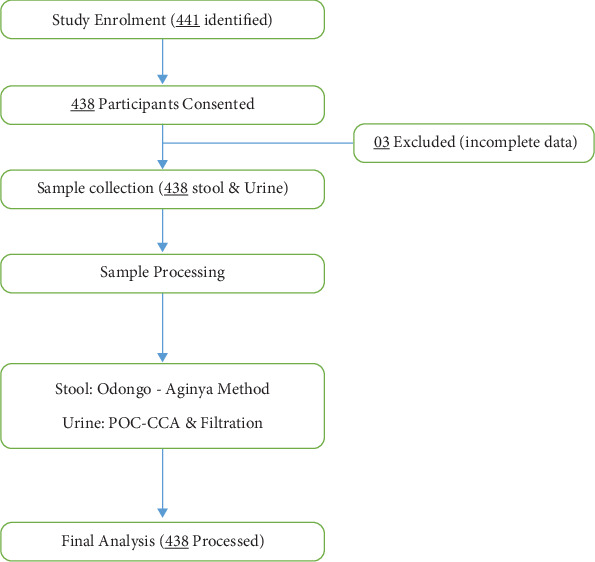
Flowchart summarizing study enrolment, sample processing, and exclusions of study participants. 03 Excluded (incomplete data)—they had missing stool and/or urine samples despite initial enrollment.

The study used a multistage sampling procedure to select representative sample sizes in the study. Three out of nine districts were selected randomly using a lottery method, followed by five subcounties, 10 parishes, and 2–3 villages. Finally, the selected villages were then surveyed for urine and stool samples obtained from 438 community members involved in water contact activities. Questionnaires were administered to the consented participants, ensuring a comprehensive understanding of the community's water use (Figure 2).

### 2.6. Stool and Urine Collection, Processing, and Examination

Only consented participants were provided with stool containers, well labeled with the participant's code, laboratory number, date, and time of sample collection. Each participant was instructed to collect an equivalent of 10 g of stool in the morning between 8 and 10 a.m. and deliver it to the laboratory within 1 h after the specimen collection. The stool specimens were processed using the Odongo-Aginya method (compound stain: consisting of 5% eosin yellow in 10% formalin mixed 1:1 with 7.5% nigrosin in 10% formalin) [34]. Cellophane cover slips cut in 25 × 40 mm pieces were presoaked in 50% glycerin [34].

Stool specimens were strained through a sieve, cleaned, and placed on a microscope slide through a template measuring 41.7 mg. A compound stain was added, and a cellophane cover slip was blotted out. The slide was then examined under a light microscope to identify and quantify *Schistosoma* eggs and other intestinal parasites based on their morphological characteristics, following WHO guidelines and using the Odongo-Aginya method [34]. Specimen processing and examination were done at the Lira Regional Referral Hospital (LRRH) laboratory, and the slides were read by two qualified technicians and re-examined by a senior laboratory technologist for quality control [34]. Urine was collected using well-labeled sterile containers with participant's ID codes. Twenty to 30 mL of freshly voided urine was collected from each participant between 10 a.m. and 2 p.m. and tested within 1 h of collection at LRRH laboratory [35]. The study used a commercial antigen test kit called point-of-care circulating cathodic antigen assay (POC-CCA) to measure the CCA levels of juvenile and adult *S. mansoni* in the urine [36]. The test was performed and reported according to the manufacturer's instructions. The tests were interpreted by lab technicians independently to accurately assess the infection prevalence [36].

The urine filtration technique was also employed to examine and quantify *S. haematobium* in urine [37] by experienced laboratory technicians in the LRRH laboratory. A 10-mL syringe with a Swinney filter holder of 13-mm diameter and polycarbonate membrane filter was employed to recover the eggs of *S. haematobium* in urine and placed on a glass slide, examined using a light microscope at ×40 objective lens [37]. Samples found to have eggs were recorded as positive and the number of eggs counted to determine the intensity (Figure 2). Those found to be positive were treated with praziquantel 40 mg/kg body weight free of charge [38].

### 2.7. Risk Factors for *Schistosoma* Infection

**Figure 3:**
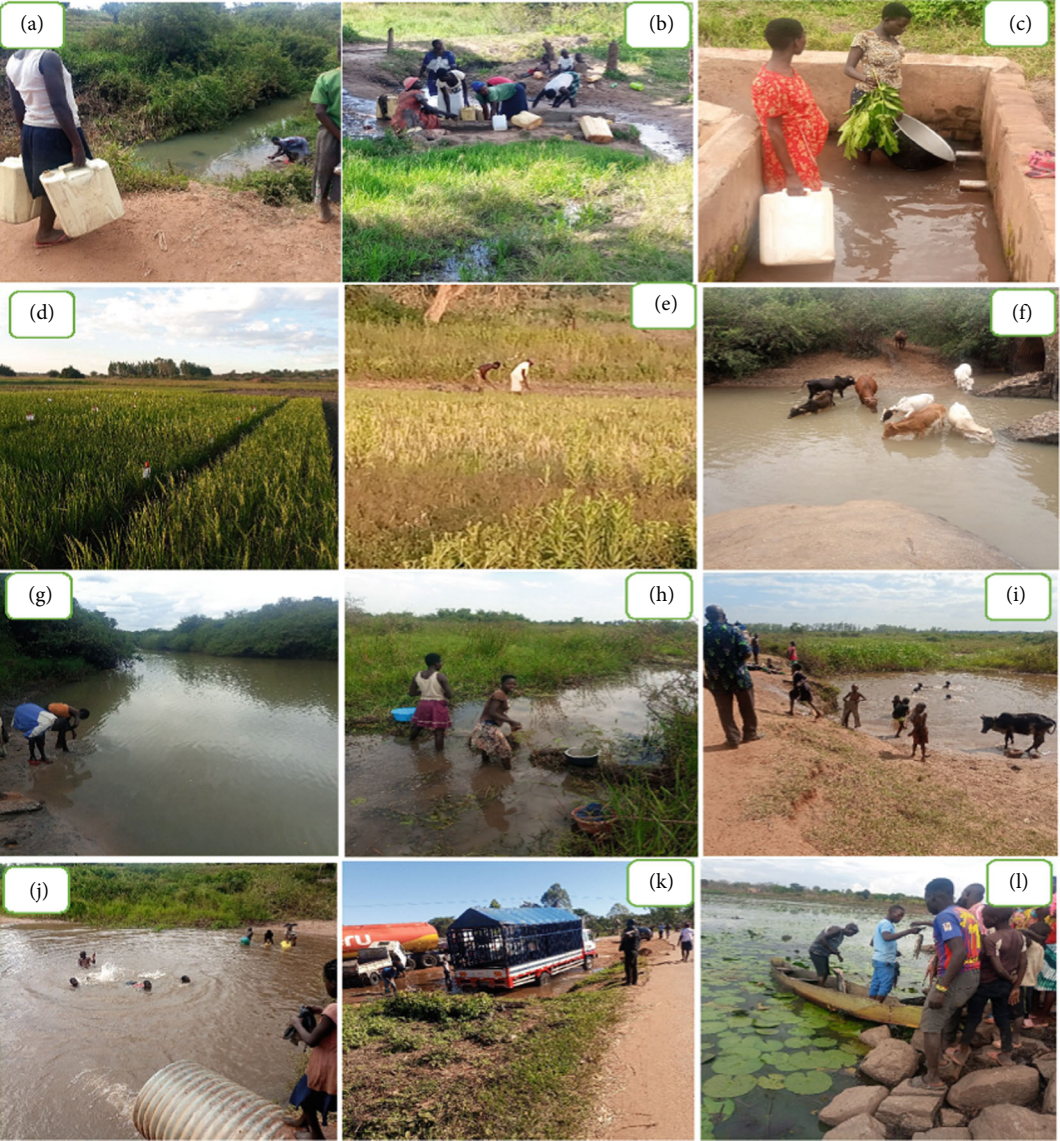
Water contact activities in the study area in the Lango subregion, northern Uganda. Fetching water (a–c), rice cultivation (d, e), grazing of cattle (f), washing and bathing (g, h), swimming (i, j), car washing (k), and fishing (l).

Consenting community members involved in any water contact activities were interviewed using a pretested questionnaire to collect the demographic and sociocultural information and other associated risk factors for *S. mansoni* infection (Figure 3). The factors investigated included education level, employment status, income level, socioeconomic activities, water contact activities, home distance from the water source, frequency, and length of exposure to an open water source and sources of domestic water.

### 2.8. Data Analysis

The data was analyzed using SPSS Version 25.0 [39]. A Pearson's chi-square test, binary, and multivariable logistic regressions were performed to assess the association between variables. The odds ratios (ORs) with 95% confidence intervals were used to measure the strength of associations. *p* values < 0.05 were considered statistically significant. The intensity of *S. mansoni* infection was calculated using WHO guidelines [37], which classify infections into light, moderate, and heavy classes.

## 3. Results

### 3.1. Sociodemographic Characteristics of Study Subjects

A total of 438 participants aged 5–85 were enrolled in the study. Out of these, 193 (44.1%) were males and 245 (55.9%) were females. The mean age of participants was 38.9 ± 15.1 years (mean ± standard deviation), with the majority, 150 (34.4%), being younger than 30 years old (Table 1). The majority of participants, 373 (85.2%), were farmers, 292 (66.7%) had a primary level of education, 234 (53.4%) lived less than 500 m from a water source, and the two most common sources of domestic water were piped water, 180 (41.1%), and boreholes, 104 (23.7%) (Figure 3). The major reasons for water contact were farming, 287 (65.5%), and fetching water for domestic use, 53 (12.1%) (Table 1). *S. mansoni* prevalence among participants was significantly associated (*p* < 0.05) with age, location, occupation, education level, source of domestic water, and contact with open water sources (Table 1).

### 3.2. Prevalence of *S. mansoni* and Other Intestinal Parasites

**Figure 4:**
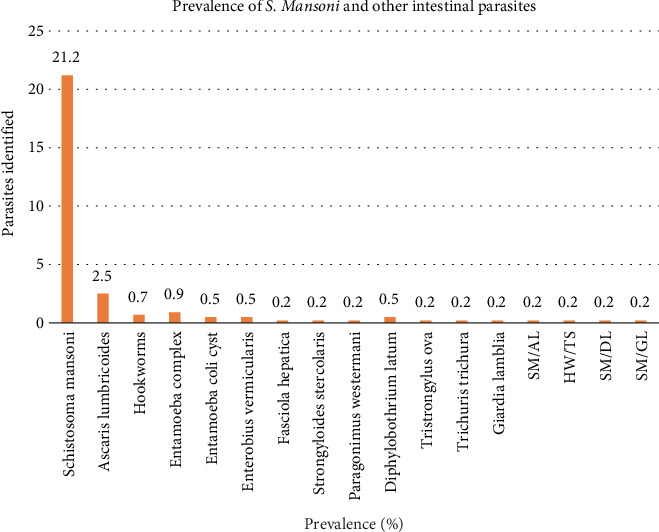
Prevalence of intestinal parasites identified in the study, *n* = 438 (pooled results POC-CCA/Odongo-Aginya method). Key: mixed infections—*Schistosoma mansoni/Ascaris lumbricoides* (SM/AL), *hookworms/Taenia* spp. (HW/TS), *Schistosoma mansoni/Diphyllobothrium latum* (SM/DL), *Schistosoma mansoni/Giardia lamblia* (SM/GL).

**Figure 5:**
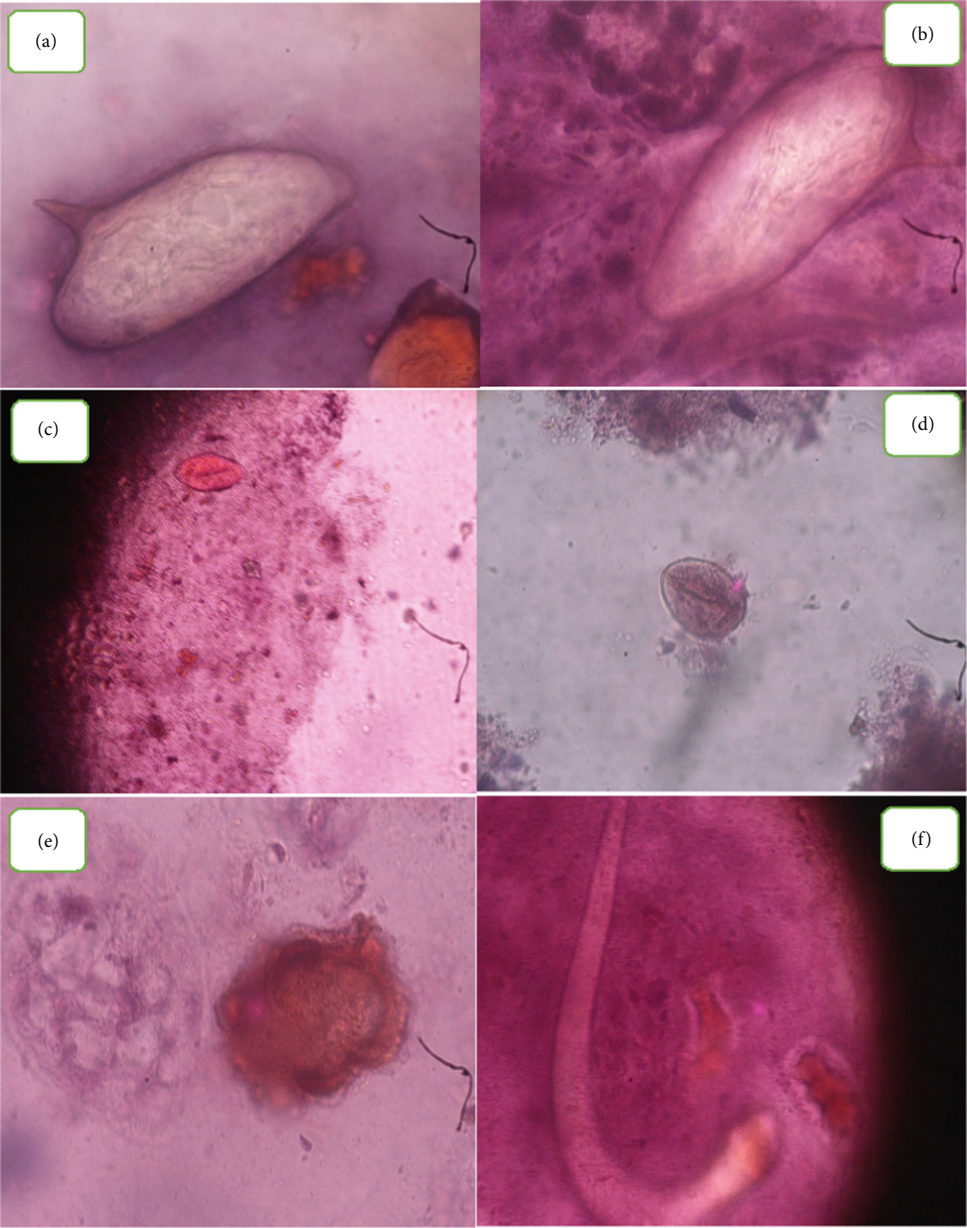
Some of the eggs and larvae of intestinal parasites identified in the study. *Schistosoma mansoni* eggs (a, b), *Enterobius vermicularis* (c, d), *Ascaris lumbricoides* (e), and *Strongyloides stercoralis* (f).

The overall prevalence of intestinal parasites including *S. mansoni* was 27.4% (120/438). *S. mansoni* had the highest prevalence of 21.2% (93/438). However, the prevalence of other intestinal parasites was low: *Ascaris lumbricoides* (2.5%); *Entamoeba complex* (0.9%); *hookworms* (0.7%); *Entamoeba coli*, *Enterobius vermicularis*, and *Diphyllobothrium latum* (*D. latum*) with 0.5%, respectively; and *Trichuris trichiura*, *Fasciola hepatica*, *Giardia lamblia*, *Strongyloides stercoralis*, *Paragonimus westermani*, and *Trichostrongylus ova* (0.2%) (Figures 4 and 5). There was a low prevalence of mixed infection with single incidences of each of *S. mansoni/Ascaris lumbricoides*, *S. mansoni/D. latum*, *S. mansoni/Giardia lamblia*, and hookworms*/Trichostrongylus ova* (Figure 4). *S. haematobium* was not detected in this study.

### 3.3. Intensity of Infection With *S. mansoni* in the Study Area

The intensity of infection was calculated based on WHO grading of eggs per gram (EPG) as light, moderate, and heavy as 1–99, 100–399, and > 400 EPG, respectively [32]. The heavy intensity of infection was found in a single female in the age group of 17–20 years with a mean egg count of 480 EPG (Table 2). The majority of the participants had low to moderate intensity of infection with mean egg counts ranging between 72–75.4 and 144–187 EPG, respectively (Table 2).

### 3.4. Risk Factors Associated With *S. mansoni* Infection

Human water contact activities in these study areas were high. All the participants visited the water bodies at least once a week, either for wetland farming, swimming, fishing, washing clothes, bathing (Figure 2), or fetching water (Table 3). In the univariable binary logistic regression, the age group of participants, subcounty location, and source of domestic water had a *p* value less than 0.05 and were further analyzed by multivariable logistic regression (Table 3). However, participants who were recruited from the subcounties of Ogur, Aromo, and Barr were 9, 6, and 4 times more likely to get infected with *S. mansoni*, respectively. Those who fish, ford, swim, and bathe in open water sources were also more likely to get infected with *S. mansoni* (Table 3). The multiple logistic analysis revealed that the only factors significantly associated with *S. mansoni* infection were the age of the participant and subcounty location (Table 3). Participants from the Aromo subcounty had two times [AOR = 1.837, 95% CI: 1.274–2.650, *p* < 0.001] higher odds of infection than those hailing from the Okwerodot subcounty. This could be due to the presence of more water bodies, specific livelihood practices, or local environmental conditions that favor snail habitats. Similarly, participants aged 5–9 years had two times [AOR = 1.448, 95% CI: 1.207–1.737, *p* < 0.001] higher odds of infection when compared to those above 50 years of age (Table 3). This could be attributed to higher exposure due to economic and domestic activities (e.g., wetland farming and water collection).

## 4. Discussion

Results indicate that there were high *S. mansoni* infections (21.2%) in the study area. This is in agreement with the study in high prevalence communities in Uganda 25.6% [40] and Gulu 20.1% [18]. However, the prevalence was lower than those reported from studies in other parts of Uganda, such as 52% in Mayuge [41] and L. Victoria fishing community, 88.6% [42]. The lower prevalence in Lango than in these other areas could be attributed to repeated MDA with praziquantel among the population of the Lango subregion [21], small sample size variations [43], or environmental conditions of the study area as compared to other studies elsewhere [15, 44].

Although *S. mansoni* was identified in this study area, all urine samples tested negative for *S. haematobium* despite previous reports pointing to the Lango subregion as harboring the parasite [4, 19, 24, 45]. This could be due to yearly mass treatment campaigns implemented by the Ministry of Health since 2003 [21]. Additionally, climate change and ecological shifts may have contributed to this finding. Climate variability affects *Schistosoma* transmission by influencing the distribution of intermediate host snails. Rising temperatures, changing rainfall patterns, and habitat degradation may have made the environment less suitable for *S. haematobium* transmission [44, 46, 47]. This aligns with findings from Codjoe et al. [44], who reported that climate variability significantly impacts the distribution of intermediate host snails, affecting schistosomiasis transmission. Similarly, McCreesh and Booth [46] demonstrated that increasing temperatures can reduce snail survival rates, leading to lower transmission of schistosomiasis. Adekiya et al. [47] further highlighted that changes in water levels and vegetation cover due to climate change have influenced *Schistosoma* transmission in sub-Saharan Africa.

Furthermore, past intervention strategies may have influenced the observed trends. Sustained MDA efforts in Uganda, particularly in high-endemic areas, have significantly reduced schistosomiasis prevalence. Adriko et al. [21] found that repeated annual praziquantel treatment led to a substantial decline in *S. mansoni* and *S. haematobium* infections over time. Similarly, Adriko et al. [48] reported that long-term MDA efforts in Uganda resulted in the near-elimination of *S. haematobium* from certain regions. Additionally, the WHO [49] emphasized that MDA, combined with water, sanitation, and hygiene (WASH) interventions, has led to significant reductions in schistosomiasis prevalence [49].

Compared to earlier research, the prevalence of other intestinal parasites was very low [50, 51]. This could be due to different interventions undertaken in the study areas, such as repeated biannual MDA of albendazole in schools and the rural communities for the past two decades and health education [19]. Previous studies have demonstrated that improved sanitation, water access, and hygiene education are critical in reducing the burden of intestinal parasitic infections [47, 52].

The intensity of infection with *S. mansoni* was studied according to WHO classification [37]. There were light, moderate, and heavy infections with *S. mansoni* among the participants with 66 (72%), 24 (26%), and 2 (2.2%), respectively. However, other studies reported higher infection intensity than the current study [42, 53]. These studies were done before praziquantel's yearly distribution to the study area's community members.

Females were more infected than males, 58.1% and 41.9%, respectively, which agrees with other studies [8, 15]. The high prevalence in females is explained by their more frequent water contact activities than the males, such as wetland farming, fetching water for domestic use, washing clothes, and bathing [15]. Similar to previous studies, we found that infection rates were higher among the 10–16 and 17–20 age groups compared to older individuals, a trend also observed in other research [15, 17]. The significance of this age group's greater involvement in economically generating activities, such as wetland farming, car and motorcycle washing, fetching water for domestic use, fishing, grazing cattle in wetlands, and also bathing in open water sources, might be infested with cercariae.

Our results showed that the age of the participant and subcounty location had significant associations with *S. mansoni* infections. The geographical location of the study areas contributed to different sources of economic activities. These therefore contributed to the infections; Lira district had the highest number of infections at 74.2%, while the subcounties of Barr (39.8%), Ogur (29.9%), and Bala (10.8%) had higher infection rates with *S. mansoni* than other subcounties (Table 1). This could be attributed to the numerous freshwater bodies in these localities compared to other study location sites. Lira district has rice irrigation schemes in Abolet, Okile, and Olilo, especially in Barr subcounty [4]. Ogur subcounty has numerous water bodies like Opeta swamp, Akano, Akangi, Obim, and Abongodyang, respectively [4]. Bala subcounty has 30% of the land surface covered with swamps like Onoo, Okwor, Alemi, Okole, Opeta, and Wang Tula, thus harboring numerous snail hosts. This finding is in agreement with previous studies elsewhere [6, 12, 15, 52]. This explains why numerous water bodies contribute to suitable habitats for the snail intermediate host and ease of transmission. Also, the frequent use of unprotected water sources for domestic use predisposes the community to the risk of infection. This concurs with a study conducted in Pader district by Omona et al. [54]. This could be due to the presence of more water bodies, specific livelihood practices, or local environmental conditions that favor snail habitats. Overall, this study highlights the impact of long-term intervention programs, climate variability, and ecological changes on schistosomiasis transmission.

## 5. Conclusions

This study highlights a high prevalence of *S. mansoni* in the Lango subregion, with age and subcounty location being major risk factors. Targeted interventions, including ongoing MDA to at-risk population groups and regions, health education, improved sanitation, and access to clean water, are essential for effective control. Community engagement and behavioral change programs can further reduce infection risks. Future research should explore the long-term effects of climate change on parasite ecology and transmission dynamics, as well as the sustainability of MDA programs in schistosomiasis-endemic areas. A multifaceted approach integrating treatment, prevention, and policy-driven strategies is crucial for sustained schistosomiasis control and eventual elimination in affected communities.

## Figures and Tables

**Table 1:** Sociodemographic characteristics of the study participants in selected areas of the Lango subregion, northern Uganda, *n* (438).

**Variables**	**Character**	**Frequency (%)**	**Positive (%)**	**p** ** value**
Gender	Male	193 (44.1)	39 (41.9)	0.641
	Female	245 (55.9)	54 (58.1)	

Age groups	5–9	4 (0.9)	2 (2.2)	< 0.001⁣^∗^
	10–16	13 (3.0)	10 (1.8)	
	17–20	31 (7.1)	12 (12.9)	
	21–30	102 (23.3)	21 (22.6)	
	31–40	99 (22.6)	14 (15.1)	
	41–50	98 (22.4)	18 (19.4)	
	> 50	91 (20.8)	16 (17.2)	

Districts	Lira district	221 (50.5)	69 (74.2)	< 0.001⁣^∗^
	Lira city	121 (27.6)	7 (7.5)	
	Kole district	96 (21.9)	17 (18.3)	

Subcounties	Aromo	17 (3.9)	4 (4.3)	< 0.001⁣^∗^
	Ogur	69 (15.8)	27 (29.0)	
	Barr	123 (28.1)	37 (39.8)	
	Agali	12 (2.7)	1 (1.1)	
	Lira	121 (27.6)	7 (7.5)	
	Bala	29 (6.6))	10 (10.8)	
	Ayer	8 (1.8)	3 (3.2)	
	Aboke	26 (5.9)	0 (0)	
	Okwerodot	33 (7.5)	4 (4.3)	

Occupation	Farmer	373 (85.2)	79 (84.9)	0.041⁣^∗^
	Employed	22 (5.0)	4 (4.3)	
	Unemployed	15 (3.4)	7 (7.5)	
	Self-employed	13 (3.0)	3 (3.2)	
	Car-washer	15 (3.4)	0 (0)	

Level of education	None	68 (15.5)	20 (21.5)	< 0.001⁣^∗^
	Primary	292 (66.7)	65 (69.9)	
	Secondary	64 (14.6)	6 (6.5)	
	Tertiary	12 (2.7)	0 (0)	
	University	2 (0.5)	2 (2.2)	

Distance of home from open water source (meters)	< 500	234 (53.4)	51 (54.8)	0.403
	500–1000	125 (28.5)	22 (23.7)	
	> 1000	79 (18.0)	20 (21.5)	

Source of domestic water	Borehole	104 (23.7)	17 (18.3)	0.689
	Dam	9 (2.1)	1 (1.1)	
	Spring	27 (6.2)	5 (5.4)	
	Stream	50 (11.4)	11 (11.8)	
	Pond	2 (0.5)	0 (0)	
	Well	66 (15.1)	15 (16.1)	
	Piped water	180 (41.1)	44 (47.3)	

History of contact with an open water source	No	5 (1.1)	3 (3.2)	0.033⁣^∗^
	Yes	433 (98.9)	90 (96.8)	

Reason for contact with an open water source	None	5 (1.1)	3 (3.2)	< 0.001⁣^∗^
	Farming	287 (65.5)	59 (63.4)	
	Bathing	26 (5.9)	12 (12.9)	
	Fording	12 (2.7)	4 (4.3)	
	Swimming	11 (2.5)	4 (4.3)	
	Fetching water	53 (12.1)	5 (5.4)	
	Grazing cattle	1 (0.2)	1 (1.1)	
	Fishing	6 (1.4)	2 (2.2)	
	Washing	37 (8.4)	3 (3.2)	

Frequency of contact with water per week	None	5 (1.1)	3 (3.2)	0.097
	1–3 times	114 (26.0)	25 (26.9)	
	More than 3 times	319 (72.8)	65 (69.9)	

Duration of contact per day	None	5 (1.1)	3 (3.2)	0.051
	< 1 h	64 (14.6)	13 (14.0)	
	1–2 h	86 (19.6)	12 (12.9)	
	> 3 h	283 (64.6)	65 (69.9)	

Family monthly income (UGX)	< 100,000	346 (79.0)	78 (83.9)	0.17
	100,000–200,000	53 (12.1)	6 (6.5)	
	> 200,000	39 (8.9)	9 (9.7)	

⁣^∗^Statistically significant figure.

**Table 2:** Intensity of *Schistosoma mansoni* infection.

**Variables**	**Character**	**Light ** **n** ** (mean egg count)**	**Moderate ** **n** ** (mean egg count)**	**Heavy ** **n** ** (mean egg count)**	**Total**
Sex	Male	15 (75.2)	4 (204)	0	19
	Female	18 (73.3)	8 (147)	1 (480)	27

Age groups (years)	10–16	4 (72)	0	0	4
	17–20	1 (72)	2 (156)	1 (480)	4
	21–30	6 (68)	5 (187.2)	0	11
	31–40	8 (78)	0	0	8
	41–50	7 (75.4)	2 (156)	0	9
	> 50	7 (75.4)	3 (144)	0	10

Total		66	24	2	92

**Table 3:** Bivariate and multivariable logistic regression analysis of possible risk factors associated with *S. mansoni* infection in selected areas of the Lango subregion, northern Uganda, during the study period.

**Variables**	**Categories**	**Binary logistic regression**		**Multivariable logistic regression**	
		**cOR (95% CI)**	**p** ** value**	**aOR (95% CI)**	**p** ** value**
Gender	Male	1.072 (0.564–2.038)	0.831		
	Female	1			

Age groups (years)	5–9	8.262 (0.454–150.185)	0.154	1.448 (1.207–1.737)	< 0.001⁣^∗^
	10–16	28.248 (3.539–225.503)	0.002⁣^∗^		
	17–20	6.592 (1.934–22.466)	0.003⁣^∗^		
	21–30	1.525 (0.623–3.731)	0.355		
	31–40	0.987 (0.384–2.541)	0.979		
	41–50	1.323 (0.531–3.300)	0.548		
	> 50	1			

Districts	Lira district	0.826 (0.061–11.133)	0.886		
	Lira city	0.545 (0.120–2.478)	0.432		
	Kole district	1			

Subcounties	Aromo	6.048 (0.375–97.658)	0.205	1.837 (1.274–2.650)	0.001⁣^∗^
	Ogur	8.652 (0.375–99.278)	0.083		
	Barr	4.020 (0.342–47.246)	0.269		
	Bala	8.043 (1.585–40.817)	0.012⁣^∗^		
	Ayer	1.557 (0.075–32.320)	0.775		
	Aboke	0	0.998		
	Okwerodot	1			

Distance of home from open water source (meters)	< 500	2.019 (0.603–6.760)	0.254		
	500–1000	2.219 (0.660–7.457)	0.197		
	> 1000	1			

Source of domestic water	Borehole	0.675 (0.263–1.731)	0.414	0.896 (0.799–1.005)	0.061
	Dam	0.148 (0.012–1.797)	0.134		
	Spring	0.303 (0.061–1.515)	0.146		
	Stream	0.230 (0.059–0.899)	0.035⁣^∗^		
	Pond	0	0.999		
	Well	0.726 (0.281–1.873)	0.508		
	Piped water	1			

Reason for contact with an open water source	Wetland farming	1.112 (0.207–5.975)	0.901		
	Bathing	2.493 (0.445–13.971)	0.299		
	Fording	5.296 (0.426–65.795)	0.195		
	Swimming	3.331 (0.382–29.084)	0.276		
	Fetching water	0.690 (0.102–4.644)	0.703		
	Grazing cattle	0	1		
	Fishing	4.238 (0.295–60.878)	0.288		
	Washing	1			

Frequency of contact with water per week	1–3 times	1			
	More than 3 times	0.664 (0.241–1.833)	0.43		

Duration of contact per day	< 1 h	1			
	1–2 h	1.410 (0.278–7.153)	0.678		
	> 3 h	1.042 (0.393–2.767)	0.934		

Family monthly income (UGX)	< 100,000	0.975 (0.297–3.199)	0.967		
	100,000–200,000	0.655 (0.155–2.770)	0.565		
	> 200,000	1			

⁣^∗^Statistically significant value.

## Data Availability

Datasets used in the analysis are available from the corresponding author upon reasonable request.

## Nomenclature

CDC: Center for Disease Control
EPG: egg per gram
GUREC: Gulu University Research Ethics Committee
IRGS: Institute of Research and Graduate Studies
LRRH: Lira Regional Referral Hospital
MEC: mean egg count
MOH: Ministry of Health
NTD: neglected tropical diseases
SAC: school-aged children
SPSS: Statistical Package for the Social Sciences
STH: soil-transmitted helminths
VCD: Vector Control Division
WHO: World Health Organization
